# The complete mitochondrial genome of *Turdus obscurus* (Passeriformes: Turdidae)

**DOI:** 10.1080/23802359.2021.1981165

**Published:** 2021-09-27

**Authors:** Huanqing Zhang, Yuehuan Dong, Guolei Sun, Qingqian Wang, Yuntao Dong, Huashan Dou, Qinguo Wei

**Affiliations:** a College of Life Science, Qufu Normal University, Qufu, China; b Hulunbuir Academy of Inland Lakes in Northern Cold & Arid Areas, Hulunbuir, China

**Keywords:** *Turdus obscurus*, mitochondrion genome, phylogenetic analysis

## Abstract

The Eyebrowed Thrush (*Turdus obscurus*) is a highly migratory bird, which breeds in northeastern Asia and overwinters in southeastern Asia. We obtained the mitochondrial genome of *T. obscurus* by Sanger sequencing. The mitogenome was 16,739 bp in length, which contains 13 protein-coding genes, 22 tRNA genes, two rRNA genes, and one control region. Its composition is consistent with the species in genus *Turdus*. Phylogenetic analysis based on the whole mitochondrial genome showed that the relationship between *T. obscurus* and *Turdus kessleri* was relatively close. This study improves the understanding of phylogeny and genetics of Turdidae and Muscicapoidea.

*Turdus obscurus* (Gmelin, 1789) is a typical *Turdus* thrush, which has a relatively small body size and is a highly migratory passerine (Brazil 2009). Adult male is rather colorful, while females and immatures are mostly grayish-brown (Clement and Hathway 2000). According to the survival status of *T. obscurus*, it is listed as 'Least Concern' in IUCN Red List (BirdLife International 2016). The tissue sample used in this study was collected from a naturally dead Eyebrowed Thrush in Hulun Lake National Nature Reserve, Inner Mongolia, China (48.3764 N, 117.5306 E). The specimen of *T. obscurus* was deposited at the Institute of Biological Resources Protection and Utilization, Qufu Normal University (www.qfnu.edu.cn, Dong Yuehuan, dongyuehuan2019@163.com) under the voucher number QFSK-56. All procedures followed the Guide to Animal Experiments of the Ministry of Science and Technology (Beijing, China), and were approved by the Qufu Normal University Institutional Animal Care and Use Committee.

The complete mitogenome of *T. obscurus* (GenBank accession number MZ337397) was sequenced to be 16,739 bp. The mitochondrial genome was sequenced in overlapping fragments from PCR products using 11 primer pairs of the mitochondrion from Dong et al. (2018). Sequences produced with Sanger technology were assembled with Mega X (Kumar et al. 2018), followed by manual correction and confirmation. The whole sequence was annotated and predicted by the online software MITOS (Bernt et al. 2013). Analysis of the genome structure for *T. obscurus* indicated that it is consistent with other Turdidae species. The mitogenome contains 37 genes, including 13 protein-coding genes (PCGs), 22 tRNAs, two rRNAs, and a control region. As in other passerine mitochondrial genome (Sun et al. 2016; Zhang et al. 2021), there is an A-T bias in the base composition of mitogenome with the following nucleotide composition: 29.29% of A, 23.47% of T, 32.27% of C, and 14.97% of G.

Following Sangster and Luksenburg (2020), we verified the identity and integrity of our mitogenome sequence of *T. obscurus* with maximum-likelihood (ML) analysis using MEGA7 of reference sequences of three commonly used markers in avian systematics: NADH dehydrogenase subunit 2 (ND2, 1041 bp; *n* = 263, incl. three of *T. obscurus*), part of cytochrome c oxidase subunit I (COI, 696 bp; *n* = 316, incl. five of *T. obscurus*), and cytochrome b (Cyt b, 1143 bp; *n* = 180, incl. four of *T. obscurus*). In each analysis, our sequence clustered with *T. obscurus*.

The complete mitochondrial genomes of 20 species in superfamily Muscicapoidea were used to explore the phylogenetic relationships. Phylogenetic analyses were reconstructed with ML using PhyloSuite v1.2.2 (Zhang et al. 2020). Each of the partitions was tested with the Akaike information criteria (AIC) in ModelFinder (Kalyaanamoorthy et al. 2017), TVM + F+R4 was selected as the best evolutionary substitution model. ML bootstrap support values were estimated using 1000 bootstrap replicates and *Passer montanus* was selected as the outgroup. The phylogenetic tree (Figure 1) characterized the phylogenetic position of *T. obscurus* within the family Turdidae. Our phylogenetic results were supported by previous studies (Voelker et al. 2007; Nylander et al. 2008; Dong et al. 2018), and the results provide insights into the phylogeny of species in Turdidae and Muscicapoidea.

**Figure 1.**
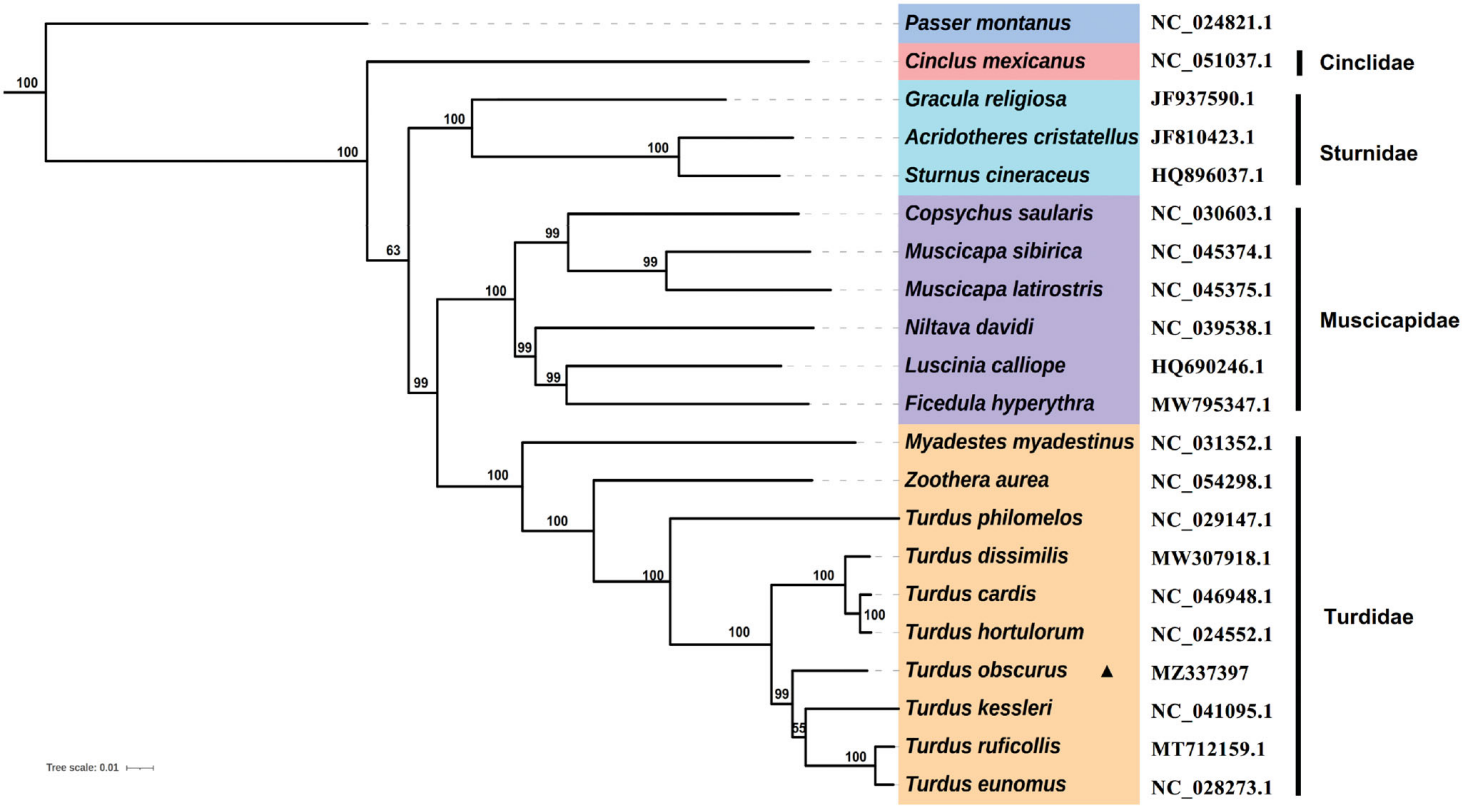
Maximum-likelihood (ML) tree of 20 species from Muscicapoidea, with an outgroup *Passer montanus*.

## Data Availability

The mitochondrial genome data that support the findings of this study are openly available in GenBank of NCBI at https://www.ncbi.nlm.nih.gov under the accession no. MZ337397.
